# How Tobacco Quitline Callers in 38 US States Reported Hearing About Quitline Services, 2010–2013

**DOI:** 10.5888/pcd13.150325

**Published:** 2016-02-04

**Authors:** Gillian L. Schauer, Ann Malarcher, Nathan Mann, Jesse Fabrikant, Lei Zhang, Stephen Babb

**Affiliations:** Author Affiliations: Ann Malarcher, Lei Zhang, and Stephen Babb, Office on Smoking and Health, National Center for Chronic Disease Prevention and Health Promotion, Centers for Disease Control and Prevention, Atlanta, Georgia; Nathan Mann, Jesse Fabrikant, RTI International, Research Triangle Park, North Carolina. Dr Schauer is contracted with the Centers for Disease Control and Prevention through Carter Consulting, Inc, Atlanta, Georgia.

## Abstract

**Introduction:**

Telephone-based tobacco quitlines are an evidence-based intervention, but little is known about how callers hear about quitlines and whether variations exist by demographics or state. This study assessed trends in “how-heard-abouts” (HHAs) in 38 states.

**Methods:**

Data came from the Centers for Disease Control and Prevention’s (CDC’s) National Quitline Data Warehouse, which stores nonidentifiable data collected from individual callers at quitline registration and reported quarterly by states. Callers were asked how they heard about the quitline; responses were grouped into the following categories: media, health professional, family or friends, and “other.” We examined trends from 2010 through 2013 (N = 1,564,437) using multivariable models that controlled for seasonality and the impact of CDC’s national tobacco education campaign, Tips From Former Smokers (Tips). Using data from 2013 only, we assessed HHAs variation by demographics (sex, age, race/ethnicity, education) and state in a 38-state sample (n = 378,935 callers).

**Results:**

From 2010 through 2013, the proportion of HHAs through media increased; however, this increase was not significant when we controlled for calendar quarters in which Tips aired. The proportion of HHAs through health professionals increased, whereas those through family or friends decreased. In 2013, HHAs occurred as follows: media, 45.1%; health professionals, 27.5%, family or friends, 17.0%, and other, 10.4%. Media was the predominant HHA among quitline callers of all demographic groups, followed by health professionals (except among people aged 18–24 years). Large variations in source of HHAs were observed by state.

**Conclusion:**

Most quitline callers in the 38-state sample heard about quitlines through the media or health care professionals. Variations in source of HHAs exist across states; implementation of best-practice quitline promotional strategies is critical to maximize reach.

## Introduction

Tobacco use is the leading preventable cause of death and disease in the United States (1). Despite decreases in cigarette smoking prevalence among adults during the past 5 decades (2), about 17% of adults smoke (3), making tobacco cessation a continued public health priority (1,2). Telephone-based tobacco cessation quitlines are an evidence-based, cost-effective tool to increase quit rates (4,5). Quitlines are available in all 50 US states, the District of Columbia, Guam, and Puerto Rico (6) and provide tobacco users with free counseling and, in many cases, free cessation medications (7).

Despite quitline effectiveness, only a small proportion of smokers use quitlines as a cessation resource (6,8). To increase the impact that quitlines have on tobacco cessation, the public health community needs to increase their reach (9). The Guide to Community Preventive Services finds that 3 approaches are effective at increasing use of quitlines: 1) mass-reach health communication interventions that include the quitline number; 2) provision of free cessation medications; and 3) use of quitline referral interventions in health care systems or by health care providers (10). These strategies are recommended to states in the Centers for Disease Control and Prevention’s (CDC’s) *Best Practices for Comprehensive Tobacco Control Programs* (11) and are part of national efforts to increase tobacco cessation. In 2012, CDC launched the first federally funded, nationwide paid-media tobacco education campaign in the United States — Tips From Former Smokers (Tips) — with the goal of increasing the number of adult smokers who quit (12). The campaign promoted 1–800-QUIT-NOW, a quitline portal that links callers to their state tobacco quitline.

An important metric in understanding and increasing quitline reach is how quitline callers report hearing about the quitline (“how-heard-abouts,” or HHAs) (13). All quitlines track HHAs or sources of referrals. Assessing changes in HHAs nationally and within states can help in evaluating the impact of tobacco control activities (eg, media campaigns, health-systems–change initiatives) and can inform efforts to increase reach (13–15).

Of the primary promotional approaches that increase quitline reach, mass-reach health communication and media campaigns (media HHAs) are the most resource intensive (16) but are highly effective nationally and in states (12,17–19). Media campaigns also generate HHAs or referrals from family or friends, who may see an advertisement or promotion and tell a friend or loved one about it (12). Generating quitline referrals from health care providers typically does not involve major promotional costs but can require a significant investment of staff time and expertise to implement systems changes, including modifying electronic health records, developing work flows, establishing quitline referral systems, training clinicians, and providing feedback on performance (14,20).

To date, no studies have gathered multistate data on quitline HHAs, although some states have used data on HHAs to monitor and evaluate components of their comprehensive tobacco control program (14,15). The objective of this study was to use data from a standardized data warehouse that collects data from multiple state quitlines to assess 1) recent trends in quitline HHAs through media, health professionals, and family or friends and 2) how HHAs vary by callers’ demographic characteristics and state. These data can be used to inform national and state planning to increase quitline reach and use.

## Methods

### Sample

Data for this study came from CDC’s National Quitline Data Warehouse (NQDW), which stores nonidentifiable data reported quarterly by state quitlines in the United States (20). We used NQDW’s individual intake data, which consists of data collected from callers during their first registration call. The NQDW intake questions were adapted from the North American Quitline Consortium’s Minimum Data Set. More information about the NQDW is available (21).

Data from the first quarter of 2010 through the last quarter of 2013 for 38 states were used for these analyses (N = 1,564,437). States that did not submit intake data to the NQDW for one or more quarters during the study period were excluded (Alaska, Colorado, Hawaii, Massachusetts, Minnesota, Montana, New Mexico, North Dakota, South Carolina, and Wyoming, plus the District of Columbia). Among these, the number of missing quarters ranged from 1 (District of Columbia) to 16 (Massachusetts). In addition, 2 states were omitted because of small sample sizes in each quarter (New Hampshire and Rhode Island). Some states allowed participants to report multiple HHAs; our analysis included only records in which participants provided only one HHA response. Records in which participants reported multiple responses were excluded (n = 31,479; 2% of the sample) because these records did not clearly indicate the primary HHA. Data from 2013 (n = 378,935) were used to assess whether HHAs varied by respondent demographics and state.

We limited our analysis to data on each unique caller per state, per quarter. However, during a single year or multiple years, data on the same caller could appear more than once; each appearance would represent a new, unique quit attempt, with a different possible quitline HHA.

### Measures

HHAs were assessed during the registration call by asking participants, “How did you hear about the quitline?” Response options included newspaper, radio, television, Internet/Web, telephone directory, flyers/brochures, health professional, family or friends, workplace, health insurance, community organizations, other, or “don’t know/not sure.” Some state quitlines may also have reported on additional HHA responses designed according to their own programs or initiatives (eg, heard about the quitline during a promotional night at a sporting event, through promotions of 1–800-QUIT-NOW on pharmacy bags). These additional response options were recoded to fit within the categories in the NQDW questionnaire.

Data collected from the NQDW were recoded into 4 larger categories: media, health professional, family or friends, and other. For this study, “media” consisted of newspapers, radio, television, Internet/Web, other media, and flyers/brochures. The “health professional” category included referrals from individual health professionals, clinics, hospitals, dental offices, health departments, The Special Supplemental Nutrition Program for Women, Infants, and Children (WIC), other clinical or health-related programs, and health insurance. Twenty-one states included health professional faxed referrals to the quitline as a separate HHA category, and these were recoded as health professional HHAs. Fax referrals were not included as a separate HHA category in 17 states. The “family or friends” category included referral from family or friends. The “other” category included community-based referrals (eg, from nonprofits, faith-based organizations, schools), former clients who were re-enrolling in the quitline, telephone directory, workplace referrals (unless part of an on-site clinic), and other miscellaneous HHAs.

Demographic data, including sex, age, race/ethnicity, and education were also collected from quitline callers. We created a binary indicator variable for quarters in which Tips aired (1 for the first and second quarters of 2012 and 2013 and 0 (zero) for all other quarters).

### Analysis

For each HHA category, we computed the frequency overall, by demographic characteristics and by state, for 2013. State data were suppressed when the number of observations was fewer than 200 for all HHA categories (New Hampshire, Rhode Island) or when an HHA category had fewer than 50 observations (Michigan, Mississippi). Because our analysis included complete data from all quitline registrants in the 38-state sample, we did not perform statistical tests for significant differences across groups.

We computed frequencies and proportions for each HHA category (media, health professional, family or friends, and other) by quarter from 2010 through 2013. We tested for trends in each HHA category using separate multivariable regression analysis by first regressing the proportions with the HHA on a linear quarterly time trend and a binary quarterly indicator variable to control for seasonality. We then included a binary indicator variable for quarters in which Tips aired. To facilitate interpretation of the regression model coefficients, we scaled the outcome variable (percentage of quitline registrants by HHA) by multiplying it by 100. Model coefficients can be interpreted as the effect of the covariate on the percentage of quitline registrants, expressed in percentage points. We plotted the predicted values after setting the Tips campaign indicator to zero for all quarters so we could observe trends after accounting for the effect of the campaign. We added trend lines to each set of predicted values. Alpha levels were set at .05 for all analyses.

## Results

**Figure 1:**
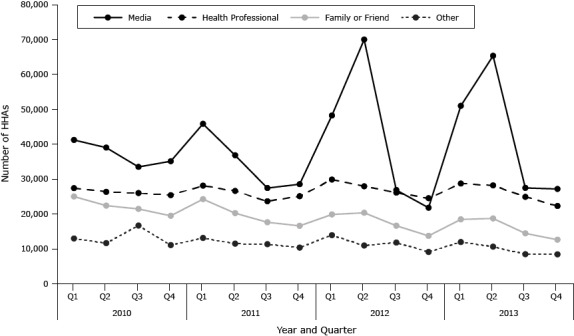
Number of quitline “how heard abouts” (HHAs) by quarter and category, 38 states, National Quitline Data Warehouse, 2010–2013. The Tips campaign aired for 12 weeks from March 19, 2012, to June 10, 2012. In 2013, the campaign was conducted for 16 weeks (10 on-air weeks and 6 off-air weeks) from March 4, 2013, to June 23, 2013. **Table:** 

In the 38-state sample, media was the most frequently reported HHA from 2010 through 2013 (Figure 1). In 2012 and 2013, peak frequencies for HHAs through media occurred in the quarters in which Tips aired (Figure 1). HHAs from health professionals, family or friends, and other sources were less variable than media HHAs. During the quarters in which Tips aired, media ranked as a larger source of HHAs than any other (eg, health professional, family or friend). 

Year	Quarter	Number of Quitline “How Heard Abouts”			
		Family or Friends	Health Professional	Media	Other
2010	1	25,026	27,388	41,208	12,977
	2	22,403	26,358	39,028	11,653
	3	21,480	25,991	33,499	16,688
	4	19,479	25,410	35,123	11,080
2011	1	24,245	28,118	45,832	13,133
	2	20,242	26,624	36,829	11,503
	3	17,627	23,645	27,450	11,346
	4	16,604	25,086	28,558	10,361
2012	1	19,876	29,873	48,224	13,937
	2	20,372	27,935	69,991	10,972
	3	16,649	26,132	26,859	11,817
	4	13,732	24,525	21,812	9,138
2013	1	18,483	28,738	50,975	11,969
	2	18,737	28,214	65,320	10,633
	3	14,448	24,907	27,479	8,480
	4	12,636	22,298	27,161	8,457

**Figure 2:**
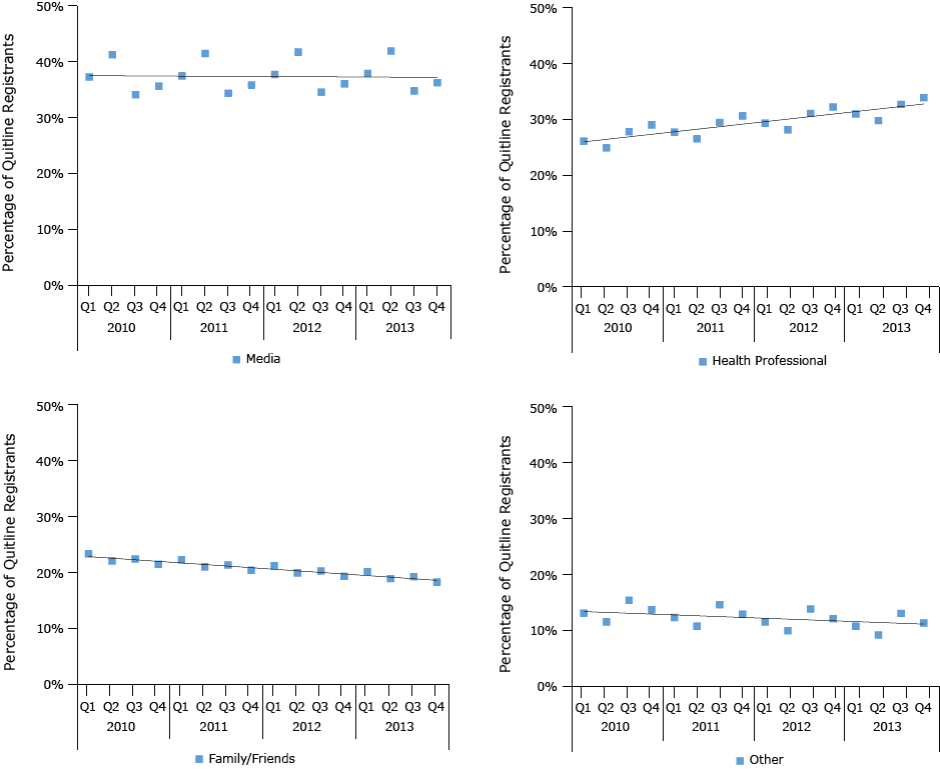
Trends in quitline registrations by “how heard about” categories, 38 states, National Quitline Data Warehouse, 2010–2013. **Table:** 

The quarterly trends indicate a significant increase in health professional HHAs (coefficient, 0.41; *P* = .02), whereas trends for family or friends HHAs (coefficient, 0.27; *P* < .001) decreased significantly from 2010 through 2013 (Figure 2). We found no significant changes in media HHAs (coefficient, 0.06; *P* = 0.81) or other HHAs (coefficient, −0.20; *P* = .06) during the study period. When the models included the Tips indicator, the media coefficient was large (9.28) and significant (*P* = .008), indicating that in quarters in which Tips aired, an additional 9.3% of quitline registrants reported hearing about the quitline from media sources. The adjusted *R* ^2^ for each model (media, health professional, family or friends, and other) ranged from 61% to 97%, suggesting that the models largely explained the variation in the percentage of quitline registrants who reported hearing about the quitline from each referral source examined.

Year	Quarter	Percentage of Quitline Registrations			
		Media	Health Professional	Family or Friends	Other
2010	1	37.3	26.1	23.4	13.1
	2	41.3	25.0	22.2	11.5
	3	34.2	27.9	22.5	15.4
	4	35.7	29.1	21.5	13.7
2011	1	37.6	27.8	22.4	12.4
	2	41.6	26.6	21.1	10.8
	3	34.4	29.5	21.4	14.6
	4	35.9	30.7	20.5	12.9
2012	1	37.8	29.4	21.3	11.5
	2	41.8	28.2	20.0	10.0
	3	34.6	31.1	20.4	13.9
	4	36.1	32.3	19.4	12.2
2013	1	38.0	31.0	20.2	10.8
	2	42.0	29.8	19.0	9.2
	3	34.8	32.8	19.3	13.1
	4	36.3	34.0	18.3	11.4

In 2013, media was the source of 45.1% of quitline HHAs in the 38-state sample, followed by health professionals (27.5%), family or friends (17.0%) and other (10.4%) (Table 1). Media was the predominant HHA category among quitline callers for all assessed demographics, followed by health professionals; the only exception was among callers aged 18 to 24 years, for whom family or friends was the second most prevalent HHA. Overall, a higher percentage of women than men reported hearing about the quitline from a health professional. The proportion hearing about the quitline from a health professional increased and the proportion hearing from family or friends decreased with increasing age. A lower percentage of non-Hispanic white callers reported hearing about the quitline from media than did the 3 other racial/ethnic groups, and a lower percentage of people of “other” non-Hispanic race/ethnicity reported hearing about the quitline from a health professional than did the 3 other racial/ethnic groups. A lower proportion of non-Hispanic black callers and callers of “other” non-Hispanic race/ethnicity reported hearing about the quitline from family or friends than did non-Hispanic white or Hispanic callers. As level of education increased, hearing about the quitline via media increased, and hearing about quitlines from a health professional decreased.

**Table 1:** Frequency of How Callers Heard About the Quitline, by Source and Demographic Characteristics, 38 States, National Quitline Data Warehouse, 2013^a^

Characteristic	Media, n (%)	Health Professional, n (%)	Family or Friends, n (%)	Other, n (%)
**Overall**	170,935 (45.1)	104,157 (27.5)	64,304 (17.0)	39,539 (10.4)
**Sex**				
Male	72,091 (46.7)	38,315 (24.8)	27,360 (17.7)	16,678 (10.8)
Female	94,371 (43.6)	63,772 (29.5)	36,092 (16.7)	22,062 (10.2)
**Age, y**				
18–24	10,700 (42.9)	5,960 (23.9)	6,080 (24.4)	2,227 (8.9)
25–44	53,271 (44.2)	30,848 (25.6)	23,847 (19.8)	12,626 (10.5)
45–64	72,635 (44.0)	49,558 (30.0)	25,244 (15.3)	17,821 (10.8)
≥65	12,074 (44.0)	8,460 (30.8)	3,864 (14.1)	3,027 (11.0)
**Race/ethnicity**				
Non-Hispanic white	97,683 (42.6)	65,097 (28.4)	42,068 (18.4)	24,249 (10.6)
Non-Hispanic black	31,824 (47.6)	18,080 (27.0)	10,107 (15.1)	6,849 (10.2)
Hispanic	10,314 (48.1)	5,621 (26.2)	3,675 (17.1)	1,831 (8.5)
Non-Hispanic other	14,991 (52.4)	6,175 (21.6)	4,464 (15.6)	2,988 (10.4)
**Education**				
<High school	27,764 (40.6)	22,898 (33.5)	11,965 (17.5)	,794 (8.5)
High school/GED	56,389 (43.7)	35,665 (27.7)	23,780 (18.4)	13,123 (10.2)
Some college	43,562 (47.0)	24,003 (25.9)	16,022 (17.3)	9,174 (9.9)
College or more	25,200 (49.3)	11,677 (22.9)	8,171 (16.0)	6,040 (11.8)

Abbreviation: GED, general educational development.

a Data were restricted to those on individuals who reported only one “how heard about” at the time of quitline intake.

Among the 38 states with available state-level data on quitline HHAs for 2013, 26 states reported media and 12 states reported health professional as the most prevalent HHA (Table 2). Only one state (Illinois) reported family or friends as the most prevalent HHA. The proportion of callers hearing about the quitline from media ranged from 14.4% (Alabama) to 66.1% (Florida); the proportion hearing about it from health professionals ranged from 10.6% (Florida) to 59.6% (Nebraska); the proportion hearing about it from family or friends ranged from 5.9% (Tennessee) to 39.8% (Illinois); and the proportion hearing about it from other sources ranged from 1.8% (Illinois) to 32.3% (New York).

**Table 2:** Frequency of How Callers in 38 US States Heard about the Quitline, National Quitline Data Warehouse, 2013^a^

State	Media, n (%)	Health Professional, n (%)	Family or Friends, n (%)	Other, n (%)
Alabama	617 (14.4)	2,448 (57.0)	474 (17.4)	484 (11.3)
Arizona	10,909 (58.9)	4,476 (24.2)	1,469 (7.9)	1,658 (9.0)
Arkansas	4,549 (34.0)	5,318 (39.8)	2,206 (16.5)	1,299 (9.7)
California	16,539 (50.2)	9,970 (30.3)	5,807 (17.6)	629 (1.9)
Connecticut	1,506 (35.8)	1,184 (28.1)	911 (21.6)	611 (14.5)
Delaware	1,094 (25.6)	1,826 (42.7)	862 (20.1)	499 (11.7)
Florida	30,353 (66.1)	4,866 (10.6)	8,123 (17.7)	2,599 (5.7)
Georgia	7,472 (57.1)	2,544 (19.4)	2,035 (15.6)	1,034 (7.9)
Idaho	1,294 (50.4)	491 (19.1)	553 (21.5)	232 (9.0)
Illinois	5,827 (35.8)	3,668 (22.5)	6,482 (39.8)	291 (1.8)
Indiana	3,883 (38.5)	3,522 (34.9)	1,494 (14.8)	1,182 (11.7)
Iowa	2,137 (24.0)	4,870 (54.7)	991 (11.1)	900 (10.1)
Kansas	1,092 (51.7)	644 (30.5)	163 (7.7)	214 (10.1)
Kentucky	1,711 (60.5)	589 (20.8)	256 (9.0)	274 (9.7)
Louisiana	2,272 (51.9)	1,039 (23.7)	681 (15.5)	389 (8.9)
Maine	1,995 (24.9)	4,314 (54.0)	1,141 (14.3)	546 (6.8)
Maryland	5,025 (51.6)	1,971 (20.2)	1,981 (20.3)	765 (7.9)
Michigan	2,449 (57.5)	1,574 (36.9)	—b	224 (5.3)
Mississippi	—b	2,342 (54.1)	1,124 (26.0)	458 (10.6)
Missouri	3,041 (54.7)	1,144 (20.6)	786 (14.1)	591 (10.6)
Nebraska	492 (22.9)	1,283 (59.6)	175 (8.1)	201 (9.3)
Nevada	437 (42.9)	257 (25.2)	166 (16.3)	159 (15.6)
New Jersey	1,820 (48.7)	695 (18.6)	699 (18.7)	524 (14.0)
New York	17,488 (44.3)	5,171 (13.1)	4,061 (10.3)	12,776 (32.3)
North Carolina	4,751 (32.4)	5,759 (39.2)	2,638 (18.0)	1,526 (10.4)
Ohio	1,828 (42.1)	1,595 (36.7)	921 (21.2)	—b
Oklahoma	9,730 (50.2)	4,771 (24.6)	3,280 (16.9)	1,588 (8.2)
Oregon	1,918 (33.3)	2,217 (38.5)	936 (16.3)	684 (11.9)
Pennsylvania	5,138 (33.8)	4,324 (28.5)	4,169 (27.5)	1,554 (10.2)
South Dakota	1,200 (28.0)	1,539 (36.0)	1,099 (25.7)	442 (10.3)
Tennessee	1,055 (62.4)	335 (19.8)	100 (5.9)	202 (11.9)
Texas	6,510 (48.5)	4,003 (29.8)	1,599 (11.9)	1,218 (9.8)
Utah	1,736 (52.7)	468 (14.2)	963 (29.2)	129 (3.9)
Vermont	485 (39.9)	447 (36.7)	217 (17.8)	68 (5.6)
Virginia	1,801 (47.8)	1,188 (31.5)	357 (9.5)	425 (11.3)
Washington	2,752 (29.9)	3,218 (35.0)	1,906 (20.7)	1,313 (14.3)
West Virginia	2,955 (29.9)	4,520 (45.8)	1,615 (16.4)	784 (7.9)
Wisconsin	4,610 (44.2)	3,404 (32.6)	1,500 (14.4)	914 (8.8)

a Among individuals reporting only one “how heard about” at the time of quitline intake.

b Data suppressed because of small sample sizes, which was defined as n <200 observations across all “how heard about” categories, or less than 50 observations in an individual “how heard about” category.

## Discussion

This is the first study to examine multistate trends in quitline HHAs over time in the United States. Major findings from this study are that media and health professionals are the top 2 HHAs overall among this 38-state sample, and with some small exceptions, across most states and demographic groups. In addition, the first federally funded nationwide paid-media tobacco education campaign, Tips, appears to have had a significant impact on media HHAs, and increases in health professional HHAs were significant even after controlling for Tips. These findings underscore the fact that cultivating both media HHAs and health professional HHAs is important because their effects can be complementary. For example, media campaigns and nicotine-replacement therapy promotions typically generate temporary spikes in quitline calls when advertisements or promotions are running (17,22,23). In contrast, if provider referrals are carefully developed, they can be a stable, ongoing source of quitline calls (20). Thus, both mass-media campaigns that generate direct calls to quitlines and health-systems–change approaches to increasing provider referrals can work in tandem to improve quitline reach. State tobacco control programs can consider prioritizing promotional programs that include both media activities and activities that integrate cessation interventions, including referral to quitlines, into health systems (11).

That health professional HHAs increased significantly over time after adjusting for the effects of the Tips campaign suggests that efforts by states and clinical and community partners to engage health professionals and systems in tobacco cessation treatment and referral have an impact on quitline use. Smokers cite clinician advice as an important motivator to try to quit, and most tobacco users visit a health professional annually, making health professionals an important referral source to connect patients with tobacco quitlines (4). Using electronic health records to refer patients to quitlines could make it even easier for clinicians to refer patients to the quitline as an adjunct to their own care or for follow-up (24).

For the most recent year of data (2013), the finding that racial/ethnic minority populations had a greater likelihood than non-Hispanic whites of reporting that they heard about the quitline from media is supported by other research showing that a higher percentage of racial/ethnic minority than white study participants remembered and responded to mass media tobacco cessation campaigns (25). In addition, Zhu and colleagues used 18 years of data from the California Smokers’ Helpline and found that African American respondents reported higher rates of media HHAs than white non-Hispanic respondents, and were more likely to call when media campaigns were airing (15). The finding that, in the 38-state sample, women were more likely than men and older respondents more likely than younger respondents to report hearing about the quitline from a health professional was probably due to the greater use of health care services by women (compared with men) and older people (compared with younger people) (26). In contrast, respondents with higher levels of education were less likely to report hearing about the quitline from a health professional and more likely to report hearing about quitlines from a media campaign than were respondents with lower educational levels. This finding may be due to some states having targeted their fax referral programs and corresponding professional educational campaigns to health care providers who serve Medicaid or other low-income populations to increase referrals for these populations (11).

HHAs also varied by state. For example, in 2013, the percentage of media HHAs varied from 14.4% in Alabama to 66.1% in Florida. Analysis by state of quitline call volume and gross rating point data from the 2012 Tips campaign indicated that Tips significantly increased quitline calls in 47 states; compared with the effect of Tips on the increase in calls nationally, the effect was larger in 11 states and smaller in 5 states (27). More research is warranted to understand state variations in HHAs and to help evaluate and inform program and resource allocation decisions that affect HHAs. For example, variation in health professional HHAs could be correlated with health-systems–change efforts in a state or health system.

This study has several limitations. First, data were self-reported and subject to inherent perception and recall bias. Second, although callers may have been exposed to multiple sources of information about the quitline, our analysis was restricted to callers who reported only one HHA. In practice, callers probably heard about the quitline in various ways, but they were permitted to indicate only the most prominent or the most recent HHA during the quitline registration call. Thus, callers not reporting family or friends as the HHA may still have heard about the quitline from family or friends. This discrepancy may explain the differences between our findings and those of McAfee et al (12), which suggested the Tips campaign led to increases in discussions about quitting between family or friends and tobacco users. Marketing theories include the idea of “effective frequency,” which posits that people require a specific frequency of exposures to a message or product before making a decision to respond (28). Behavioral theories, including the Elaboration Likelihood Model of Persuasion (29) and the Theory of Planned Behavior (30), suggest that the way the message is framed and presented and who delivers the message can affect how people process the message and act on the recommended behavior. Therefore, delivering information about the quitline through numerous types of people (eg, family and friends, health professionals) and through various modes (ie, from personal conversations to mass media) may be an effective approach. These data do not capture the extent to which other messages or sources of messages may have affected the individual’s decision to contact the quitline. Third, for states collecting fax referral information, we recoded fax referrals as health care professional HHAs. To determine whether or not responses coded as fax referrals were actually health professional HHAs, we conducted a secondary analysis in 3 large states (Florida, Texas, and North Carolina) that provided data on both fax referrals and health professional HHAs and found a high concordance (68%–83%) between people who were referred by fax and people who reported they heard about the quitline from a health professional. Fourth, these data do not represent all 50 states but rather a 38-state sample. A study sample that included data from all states and territories may have had different findings. Finally, although we controlled for calendar quarters in which CDC’s national Tips campaign aired, we did not control for paid or earned media activities conducted by state tobacco control programs separately from Tips because we did not have access to those data. Furthermore, the quarters analyzed did not exactly match the quarters in which Tips aired; Tips did not run in all weeks of all quarters examined.

Our data suggest that media and health professionals were the 2 most commonly reported sources for hearing about the quitline in our 38-state sample. CDC’s Tips campaign significantly affected media HHAs when the campaign aired, and the proportion of referrals from health professionals increased from 2010 through 2013 after adjusting for the campaign. In accordance with CDC’s *Best Practices for Comprehensive Tobacco Control Programs* (11), state tobacco control programs should support both media and health-systems–change efforts to diversify the sources of quitline calls and reach a broader pool of callers.
